# Dual-Responsive Nanoparticles for Prodrug Activation and Copper-Dependent Cell Death

**DOI:** 10.3390/nano16201301

**Published:** 2026-10-14

**Authors:** Changwen You, Yongming Wang, Qihong Zhang, Liyin Ye, Xinhong Wang, Zhenfei Peng

**Affiliations:** 1 School of Medical Technology and Engineering, Fujian Health College, Fuzhou 350101, China; 15205914073@163.com (Q.Z.);; 2 College of Biological Science and Engineering, Fuzhou University, Fuzhou 350108, China; 210820020@fzu.edu.cn; 3 Department of Pathology, Guangzhou Medical University, Guangzhou 510182, China

**Keywords:** stimulus-responsive release, oxidative stress, cuproptosis, in situ drug formation, metal–polyphenol networks

## Abstract

Cuproptosis represents a promising strategy for cancer therapy. Generating copper diethyldithiocarbamate (Cu(DTC)_2_) directly at tumor sites may enhance copper-dependent antitumor activity; however, inefficient precursor loading and premature release may limit local Cu(DTC)_2_ formation and increase off-target toxicity. To overcome these challenges, a reactive oxygen species (ROS)-responsive, thioketal-linked dopamine dimer (DTD) and an H_2_O_2_-responsive DTC prodrug (DQ) were co-assembled and subsequently coated with a copper–tannic acid (Cu@TA) network to form CDD nanoparticles (CDD NPs). The DQ-loading capacity and stimulus responsiveness of CDD NPs were characterized, and their in vitro anticancer activity was evaluated in 4T1 cells. DTD promoted DQ loading and linked the core to the copper network through catechol coordination. DTD-free CD NPs contained only 57.3% of the DQ in CDD NPs and had a lower Cu(I) fraction. Copper release from the Cu@TA network was limited under physiological conditions but increased in an acidic environment, whereas H_2_O_2_ triggered DQ cleavage, supporting the proposed in situ formation of Cu(DTC)_2_. In 4T1 cells, CDD NPs induced oxidative stress, glutathione depletion, and apoptosis. Intracellular copper accumulation, partial rescue by tetrathiomolybdate, and changes in ferredoxin 1 (FDX1) and dihydrolipoamide S-acetyltransferase (DLAT) suggested possible cuproptosis involvement. CDD NPs improved prodrug loading and controlled precursor release, inhibiting tumor cells in vitro.

## 1. Introduction

The discovery of cuproptosis, a form of regulated cell death driven by intracellular copper accumulation, has renewed interest in copper-based cancer therapy [1,2,3]. In this process, excess copper binds directly to lipoylated proteins in the tricarboxylic acid cycle, causing their aggregation and the loss of iron–sulfur cluster proteins [4]. Copper also participates in Cu^2+^/Cu^+^ redox cycling [5,6,7]. Cu^2+^ can oxidize glutathione (GSH), while Cu^+^ reacts with hydrogen peroxide (H_2_O_2)_ to generate highly reactive hydroxyl radicals (•OH) through Fenton-like reactions [8,9,10]. However, insufficient intracellular copper can limit the efficacy of cuproptosis-based therapy [11]. Direct administration of copper salts may also cause nonspecific copper accumulation and toxicity [12,13]. Thus, controlled delivery and intracellular release of copper are important for improving copper-based tumor therapy [14,15].

Disulfiram (DSF), a clinically approved treatment for alcohol dependence, has been reported to exert anticancer effects when combined with copper [16,17]. Its metabolite, diethyldithiocarbamate (DTC), readily chelates copper to form cytotoxic copper diethyldithiocarbamate (Cu(DTC)_2_), which disrupts cellular redox balance and protein homeostasis [18,19]. However, DSF is unstable in the bloodstream, and its rapid degradation limits the formation of Cu(DTC)_2_ at tumor sites [19]. Direct administration of Cu(DTC)_2_ is also limited by poor stability and a lack of tumor selectivity [19]. H_2_O_2_-responsive DTC prodrugs provide a possible solution by protecting DTC during delivery and allowing Cu(DTC)_2_ to form after activation [20]. Co-delivery of copper and DTC precursors has been explored to facilitate Cu(DTC)_2_ formation for cancer treatment [21].

Nanocarriers can combine metal-mediated chemodynamic therapy with chemotherapy and photothermal treatment within a single formulation [22]. For copper/DTC-based therapy, nanocarriers have been developed to reduce premature drug exposure and improve the co-delivery of copper and DTC-based agents [23]. Nevertheless, conventional carrier-based systems may suffer from low drug loading, premature leakage, and complex preparation [24]. Small-molecule co-assembly can improve the incorporation of hydrophobic drugs without requiring a large amount of inert carrier material [25,26,27]. Metal–polyphenol coordination networks provide another useful approach because they can retain copper at neutral pH and release it when the coordination bonds weaken under acidic conditions [28,29,30]. However, effective incorporation of hydrophobic drugs into metal–polyphenol network (MPN)-based formulations depends on drug–matrix interactions and assembly conditions [29,31]. These considerations motivated the use of a coordination-active co-assembly component to promote DQ incorporation within the copper-containing formulation. A molecule capable of participating in both drug co-assembly and copper coordination could connect the prodrug directly with the copper-containing network, thereby improving prodrug incorporation and retaining both reaction partners in one formulation [32].

Herein, CDD NPs were constructed by integrating DTD/DQ co-assembled nanoassemblies (DD NPs) with a copper-tannic acid (Cu@TA) coordination network. The amphiphilic, reactive oxygen species (ROS)-responsive dimer DTD co-assembled with hydrophobic DQ, while its catechol groups provided additional coordination sites for Cu^2+^. DTD thus acted as a coordination-active co-assembly component that connected the DQ-containing core to the copper-bearing network. This structural role extends beyond improved DQ loading by integrating prodrug incorporation and copper retention within the same nanoparticle. The design combines acid-responsive copper release with H_2_O_2_-responsive DQ activation, providing a basis for the proposed intracellular formation of Cu(DTC)_2_. The experiments demonstrated stimulus responsiveness in cell-free systems and copper-dependent cytotoxicity accompanied by oxidative stress and apoptosis in cultured cells. They also suggested a contribution from cuproptosis.

## 2. Materials and Methods

### 2.1. Materials and Reagents

Reduced glutathione (GSH) and tannic acid (TA) were supplied by Yuanye Bio-Technology Co., Ltd. (Shanghai, China). Trifluoroacetic acid (TFA) and 3-mercaptopropionic acid (MPA) were purchased from Energy Chemical (Shanghai, China). Sinopharm Chemical Reagent Co., Ltd. (Shanghai, China) provided anhydrous acetone and N-hydroxysuccinimide (NHS). 4-(Bromomethyl)benzeneboronic acid pinacol ester, sodium diethyldithiocarbamate trihydrate, dopamine (DA), and fluorescein isothiocyanate (FITC) were purchased from Aladdin (Shanghai, China). Sodium hydroxide (NaOH), 30% hydrogen peroxide (H_2_O_2_), ethanol (EtOH), methanol (MeOH), copper(II) chloride dihydrate (CuCl_2_·2H_2_O) and ethyl acetate (EtOAc) were supplied by Sigma-Aldrich (St. Louis, MO, USA). Roswell Park Memorial Institute (RPMI) 1640 medium, phosphate-buffered saline (PBS), 0.25% trypsin–EDTA and Dulbecco’s modified Eagle’s medium (DMEM) were purchased from Gibco (Thermo Fisher Scientific, Waltham, MA, USA). Biosharp (Hefei, China) provided fetal bovine serum (FBS) and the Cell Counting Kit-8 (CCK-8). Calcein sodium salt, 5,5′-dithiobis(2-nitrobenzoic acid) (DTNB), ammonium tetrathiomolybdate (TTM), and 3,3′,5,5′-tetramethylbenzidine (TMB) were supplied by Macklin (Shanghai, China). Beyotime Biotechnology (Shanghai, China) supplied calcein acetoxymethyl ester (calcein-AM), 4′,6-diamidino-2-phenylindole (DAPI), propidium iodide (PI), and the Annexin V-FITC apoptosis detection kit, 2′,7′-dichlorodihydrofluorescein diacetate (DCFH-DA), and GSH assay kits. Antibodies against caspase-3 (A83323), BCL2-associated protein (BAX; A38181), dihydrolipoamide S-acetyltransferase (DLAT; A61660), and ferredoxin 1 (FDX1; A72860) were purchased from Nature Biosciences (Hangzhou, China).

### 2.2. Synthesis of CDD NPs

Synthesis of DQ: DQ was synthesized according to a previously reported procedure [18]. 4-(Bromomethyl)benzeneboronic acid pinacol ester (0.50 g, 1.68 mmol) was dissolved in tetrahydrofuran (10 mL). This solution was slowly introduced at room temperature into sodium diethyldithiocarbamate trihydrate (3.80 g, 16.85 mmol) dissolved in methanol (10 mL) under stirring. After overnight stirring, the solvents were removed under vacuum, and the residue was taken up in ethyl acetate. The organic solution was washed first with water and then with brine, dried with anhydrous Na_2_SO_4_, and filtered. After concentration and purification by silica gel column chromatography, DQ was obtained as a white solid.

Synthesis of DTD: The ROS-responsive dimeric prodrug dopamine dimer DTD was synthesized through an amidation reaction according to a previously reported method [33]. Briefly, the thioketal-containing linker (TK) (0.4 mmol) was activated with NHS (0.8 mmol) and 1-ethyl-3-(3-dimethylaminopropyl)carbodiimide hydrochloride (EDC·HCl) (0.8 mmol) in dry N,N-dimethylformamide (DMF) (3 mL) at 4 °C under nitrogen for 30 min. DA (0.9 mmol) dissolved in anhydrous DMF (2 mL) was then added dropwise, and the mixture was stirred at room temperature in the dark for 24 h. DTD was collected by precipitation with cold diethyl ether, washed three times, and dried under vacuum.

Synthesis of DD NPs: Separate dimethyl sulfoxide (DMSO) solutions of DTD and DQ were prepared at 40 and 10 mg mL^−1^, respectively. The DTD solution (0.50 mL, 20 mg) was combined with the DQ solution (0.25 mL, 2.5 mg). The resulting mixture was gradually added to 20 mL of deionized water while stirring at 25 °C. Stirring was continued for 60 min to produce DD NPs by solvent displacement.

Synthesis of CDD NPs: DD NPs were dissolved in 40 mL of deionized water. An aqueous solution containing CuCl_2_·2H_2_O (12.8 mg) was introduced gradually while the suspension was stirred. Five minutes later, an aqueous TA solution (10 mg) was slowly introduced, and 0.1 M NaOH was used to raise the pH to approximately 8.0. The suspension was stirred at room temperature for 12 h, allowing the Cu@TA coating to form. CDD NPs were recovered by centrifugation, rinsed three times, and stored either as an aqueous dispersion or as a lyophilized powder.

### 2.3. Nanoparticle Characterization

The morphology of CDD NPs was examined by transmission electron microscopy (TEM; JEM-2100, JEOL Ltd., Akishima, Japan). Their hydrodynamic diameter and zeta potential were measured using a BeNano 90 Zeta analyzer (Bettersize Instruments Ltd., Dandong, China). Surface elemental composition and the chemical states of Cu were analyzed by X-ray photoelectron spectroscopy (XPS; K-Alpha, Thermo Fisher Scientific, Waltham, MA, USA). X-ray diffraction patterns were recorded using an Ultima IV diffractometer (XRD; Rigaku Corp., Akishima, Japan) to evaluate crystallinity. Fourier-transform infrared (FTIR) and ultraviolet–visible (UV–vis) spectra were obtained using a Spectrum Two spectrometer (PerkinElmer, Shelton, CT, USA) and a UV-2600 spectrophotometer (Shimadzu Corp., Kyoto, Japan), respectively. ^1^H and ^13^C nuclear magnetic resonance (NMR) spectra were recorded on a Bruker 400 MHz NMR spectrometer (Bruker BioSpin, Rheinstetten, Germany). Fluorescence images were captured with a CKX53 inverted fluorescence microscope (Olympus, Hachioji, Japan). Calcein fluorescence intensity and CCK-8 absorbance at 450 nm were measured with a microplate reader (BioTek Instruments, Winooski, VT, USA).

### 2.4. In Vitro Cu Release and DQ Loading Assays

CDD NP dispersion (5 mL) was enclosed in a dialysis bag with a molecular weight cutoff of 10 kDa. The bag was immersed at 37 °C in PBS at pH 7.4, 6.5, or 5.5, or in PBS at pH 5.5 supplemented with 1 mM H_2_O_2_. At each sampling interval, a 1 mL aliquot was withdrawn from the external medium and immediately replenished with fresh medium of the same composition. The copper content in the collected samples was measured by inductively coupled plasma (ICP) analysis, and cumulative copper release was calculated as a percentage of the total copper content in CDD NPs.

DQ loading in CDD NPs and DTD-free CD NPs was determined by ICP analysis of B, which was derived exclusively from DQ. A known amount of each sample was digested with aqua regia and diluted to a defined volume before analysis. DQ loading was calculated from the measured B content and the stoichiometric B content of DQ. For comparison, the DQ loading in CDD NPs was normalized to 100%, and the relative loading in CD NPs was calculated accordingly.

### 2.5. H_2_O_2_-Responsive Behavior of DQ

To follow the reaction of DQ with H_2_O_2_, DQ (10 mg) was dissolved in DMSO-d_6_/D_2_O (900/100 μL) in an NMR tube. After H_2_O_2_ (30 μL, 30 wt%) was added, ^1^H NMR spectra were acquired at predetermined time points.

H_2_O_2_-dependent DQ activation was also assessed indirectly by calcein–Cu^2+^ competition. Calcein and CuCl_2_·2H_2_O were mixed in PBS, which quenched calcein fluorescence. The final concentrations of H_2_O_2_, DQ, calcein, and Cu^2+^ were 125, 12.5, 1, and 1 μM. DQ was added, and samples with or without H_2_O_2_ were run in parallel. Fluorescence recovery was used as an indirect indicator of competition for Cu^2+^ by DQ-derived products, consistent with the proposed formation of Cu(DTC)_2_. Fluorescence recovery was measured at predetermined intervals with excitation at 485 nm and emission at 528 nm. The reaction mixture was subsequently analyzed by mass spectrometry.

### 2.6. Evaluation of H_2_O_2_-Dependent Oxidative Activity

H_2_O_2_-dependent oxidative activity of CDD NPs was examined by monitoring the oxidation of TMB. Oxidized TMB (oxTMB) produces a blue color with a characteristic absorption peak at approximately 652 nm. CDD NPs were incubated with TMB and H_2_O_2_ in PBS at pH 5.5, followed by measurement of the UV–vis absorption spectra over 400–800 nm. DQ, TA, and CDD NPs at pH 7.4 were examined under otherwise identical conditions for comparison. Concentration-dependent TMB oxidation generation was evaluated using CDD NP concentrations of 12.5, 25, 50, 100, and 200 μg mL^−1^. For the time-dependent experiment, spectra were acquired at 0, 30, 60, 90, and 120 min while all other conditions were kept constant.

### 2.7. In Vitro GSH Consumption Assay

GSH consumption by CDD NPs was quantified using DTNB. Equal volumes of GSH (1 mM) solution in PBS (pH 7.4) and CDD NP dispersion were incubated at 37 °C. Time-course samples were collected at 0, 15, 30, 60 and 90 min. In a separate experiment, CDD NPs were tested at 0, 7, 14, 21, 28 and 35 μg mL^−1^ and incubated for 30 min. Residual GSH was reacted with DTNB and measured at 412 nm. GSH content was normalized to the untreated control.

### 2.8. Cell Culture

The murine breast cancer cell line 4T1, the Lewis lung carcinoma (LLC) cell line and NIH 3T3 cells were purchased from the Cell Bank of the Chinese Academy of Sciences (Shanghai, China). RPMI 1640 supplemented with 10% FBS and 1% penicillin–streptomycin was used for 4T1 cells. LLC cells and NIH 3T3 were grown in DMEM containing the same supplements. All three cell lines were kept in a humidified incubator at 37 °C under 5% CO_2_.

### 2.9. In Vitro Cytotoxicity and TTM Rescue Assays

The 4T1 and LLC cells were plated in 96-well plates at 5 × 10^3^ cells per well. Following overnight attachment, the cells were incubated with CDD NPs at 0, 0.125, 0.25, 0.5, 1, or 2 μg mL^−1^ for 24 or 48 h. For comparisons among different formulations, 4T1 cells were exposed to the indicated concentrations of TA, DQ, Cu@TA, or CDD NPs for 24 h. CCK-8 solution was subsequently introduced into each well at 10% (*v*/*v*), followed by incubation for 30 min. Absorbance was then read at 450 nm, with the untreated group serving as the reference for relative cell viability.

The cytotoxicity of tetrathiomolybdate (TTM) alone was examined at 25, 50, 100, and 200 μg mL^−1^. For the rescue assay, 4T1 cells were treated with CDD NPs (0.2–1 μg mL^−1^) in the absence or presence of TTM (20 μM). The effects of TTM on Cu@TA- and CuCl_2_-induced cytotoxicity were tested in parallel. Cell viability was measured using the same CCK-8 procedure. Rescue experiments at higher CDD NP concentrations were conducted under identical conditions.

### 2.10. Wound Healing Assay

The 4T1 cells were grown to form confluent monolayers in six-well plates. A sterile 200 μL pipette tip was drawn through each monolayer to create a cell-free gap. Dislodged cells were cleared by rinsing with PBS, after which the cultures received fresh RPMI 1640 containing TA, DQ, Cu@TA, or CDD NPs. Images of the gaps were captured with an inverted microscope at 0, 24, and 48 h. ImageJ was used to calculate the relative migration area. All scratches were made by the same operator using sterile 200 μL pipette tips guided along a straight reference line. Each treatment group included three wells. Wound areas were measured using ImageJ, and wound closure was calculated as (A_0_ − A_t_)/A_0_ × 100%, where A_0_ and A_t_ denote the wound areas at 0 h and the indicated time point, respectively.

### 2.11. Measurement of Intracellular ROS

DCFH-DA served as a nonspecific fluorescent probe of intracellular oxidation. After attachment, 4T1 cells were exposed to TA, DQ, Cu@TA, or CDD NPs (5 μg mL^−1^) for 24 h. The cells were rinsed with PBS and incubated with DCFH-DA for 30 min according to the manufacturer’s instructions. After excess probe was removed, fluorescence images were recorded under an inverted fluorescence microscope. Fluorescence intensity was quantified from three images per group. The quantitative results are presented as mean ± SD (*n* = 3) in Figure S13.

### 2.12. Measurement of Intracellular GSH

Intracellular GSH levels were measured using a commercial GSH assay kit. The 4T1 cells were cultured overnight in six-well plates and then exposed to fresh medium alone (control), TA, DQ, Cu@TA, or CDD NPs at 5 μg mL^−1^ for 24 h. After treatment, the cells were washed with PBS and processed according to the kit protocol. GSH content was expressed relative to that of the untreated control.

### 2.13. Cell Apoptosis Assay

The 4T1 cells were plated in six-well plates at 2.0 × 10^5^ cells per well and allowed to attach overnight. The cells were exposed to PBS (control), DQ, Cu@TA, or CDD NPs at 5 μg mL^−1^ for 16 h. At the end of treatment, the cultures were rinsed three times with PBS and harvested by trypsinization and centrifugation. The collected cells were labeled with Annexin V-FITC and PI following the kit protocol and examined by flow cytometry. Unstained cells were used to set detector voltages/gains, and fluorescence compensation was performed in FlowJo using Annexin V–FITC-only and PI-only controls. All samples were acquired under identical detector settings and analyzed using the same compensation matrix.

### 2.14. Western Blot Analysis

The 4T1 cells were seeded in six-well plates and cultured overnight. Cells received DQ, Cu@TA, or CDD NPs (10 or 20 μg mL^−1^) for 24 h. Total protein was extracted with radioimmunoprecipitation assay (RIPA) lysis buffer, and protein concentrations were determined by the bicinchoninic acid (BCA) assay. Equal amounts of protein were separated by sodium dodecyl sulfate–polyacrylamide gel electrophoresis (SDS-PAGE) and transferred onto polyvinylidene difluoride (PVDF) membranes. After blocking, the membranes were incubated with primary antibodies against caspase-3, cleaved caspase-3, BAX, FDX1, DLAT, and α-tubulin. The corresponding horseradish peroxidase (HRP)-conjugated secondary antibodies were subsequently applied. Protein bands were developed with an enhanced chemiluminescence reagent and recorded with a gel-imaging system. α-Tubulin served as the loading control.

### 2.15. Hemolysis Assay

Fresh mouse erythrocytes were washed three times with PBS and diluted to obtain a 2% erythrocyte suspension. The erythrocytes were incubated with CDD NPs at final concentrations of 0, 5, 10, 25, 50, and 200 μg mL^−1^ at 37 °C for 2 h. PBS and deionized water were used as the negative and positive controls, respectively. After centrifugation, the absorbance of the supernatant was measured at 540 nm, and the hemolysis rate was calculated relative to the positive and negative controls. All measurements were performed in triplicate.

### 2.16. Statistical Analysis

All data were presented as mean ± standard deviation and analyzed using GraphPad Prism 7.0. Statistical significance was assessed by one-way analysis of variance (ANOVA): * *p* < 0.05, ** *p* < 0.01, *** *p* < 0.001, **** *p* < 0.0001; ns, not significant.

## 3. Results

### 3.1. Preparation and Characterization of CDD NPs

**Figure 1:**
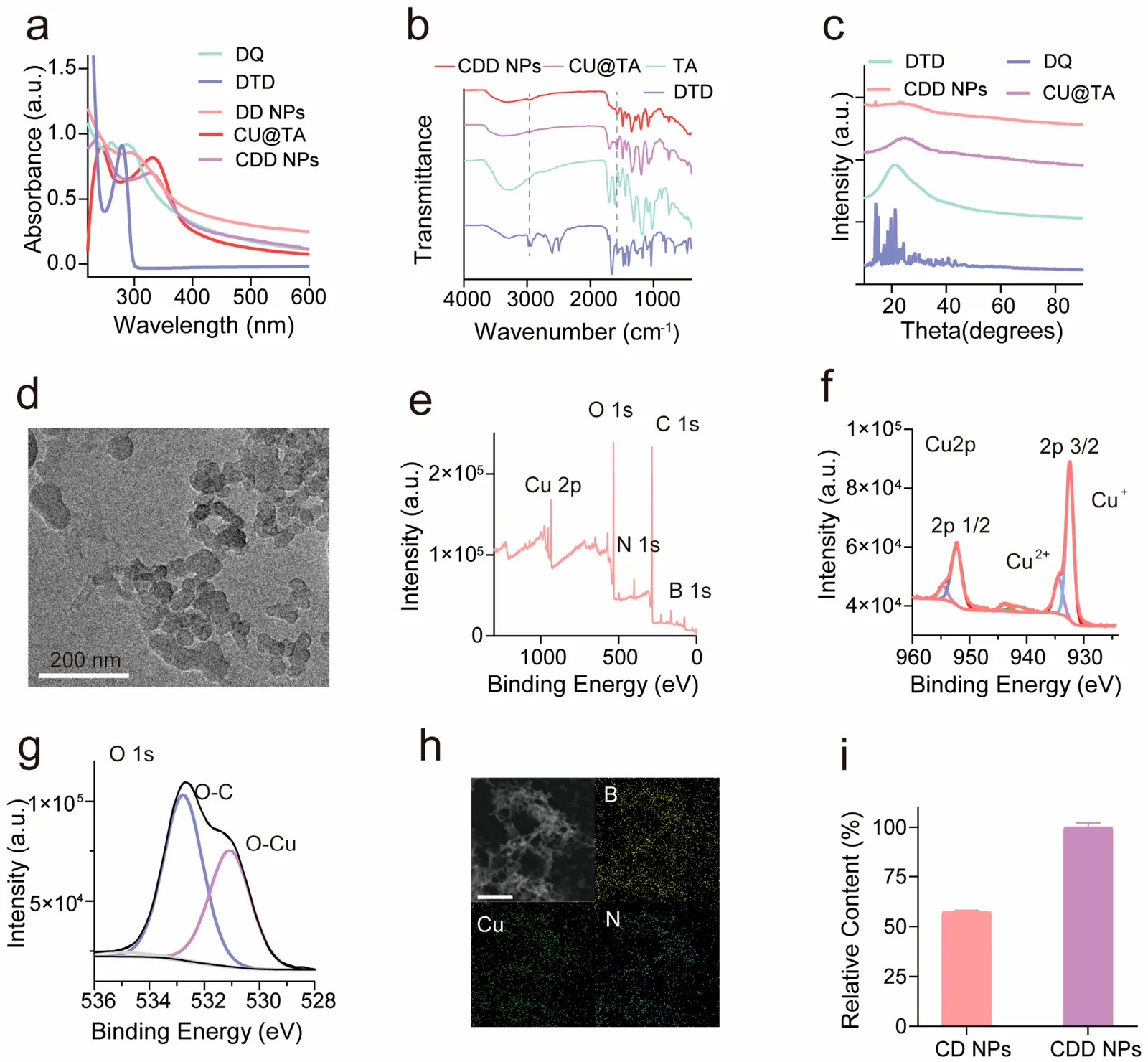
Preparation and characterization of CDD NPs. (**a**) UV–vis spectra of DQ, DTD, DD NPs, Cu@TA, and CDD NPs; (**b**) FTIR spectra of DTD, TA, Cu@TA, and CDD NPs; (**c**) XRD patterns of DQ, DTD, Cu@TA, and CDD NPs; (**d**) CDD NP morphology observed by TEM (scale bar: 200 nm); (**e**) XPS spectrum of CDD NPs; (**f**,**g**) high-resolution Cu 2p and O 1s spectra; (**h**) elemental mapping of B, Cu, and N (scale bar, 250 nm); (**i**) relative DQ loading in CD NPs and CDD NPs determined by ICP analysis of B.

CDD NPs were fabricated by coating DTD/DQ co-assembled nanoparticles (DD NPs) with a Cu@TA coordination network. The H_2_O_2_-responsive prodrug DQ and the thioketal-containing, ROS-responsive dimer DTD were synthesized according to reported methods, and their structures were confirmed by NMR (Figures S1–S3) [18,33]. The hydrophilic DA moieties and hydrophobic TK linker rendered DTD amphiphilic, allowing it to co-assemble with hydrophobic DQ to form DD NPs. The catechol groups of DTD also coordinated Cu^2+^ and linked the DD NPs to the Cu@TA network, yielding CDD NPs. To assess the role of DTD in DQ incorporation, DQ was incorporated into Cu@TA without DTD. ICP-MS analysis of B showed that DQ loading in the DTD-free nanoparticles was only 57.3% of that in CDD NPs (Figure 1i), which contained 6.70 ± 0.17 wt% DQ and 26.71 ± 0.17 wt% copper (Table S1). The lower loading in the DTD-free group supports the role of DTD in promoting DQ incorporation. DTD therefore served as both a co-assembly component and a coordination link between the DD NPs and the Cu@TA network.

The assembly of CDD NPs was first verified by UV–vis spectroscopy (Figure 1a). DTD and DQ showed characteristic peaks at 278 nm and at 260 and 286 nm, respectively. After co-assembly, DD NPs displayed a broader absorption band covering these regions. This broadening indicated a change in the molecular environment of DTD and DQ. Cu@TA showed peaks at 242 and 332 nm. In CDD NPs, the corresponding peaks shifted to 239.5 and 327 nm and became broader. These spectral changes support the incorporation of DD NPs into the Cu@TA coordination network.

FTIR spectroscopy was then used to examine the coordination of Cu^2+^ with phenolic groups (Figure 1b). DTD showed a characteristic C-H stretching band at 2940 cm^−1^, and a corresponding band appeared at 2926 cm^−1^ in CDD NPs. TA showed bands at 1608 and 1180 cm^−1^, assigned to aromatic C=C and phenolic C–O stretching vibrations, respectively. In Cu@TA, these bands appeared at 1578 and 1192 cm^−1^; in CDD NPs, they appeared at 1574 and 1194 cm^−1^. Both Cu@TA and CDD NPs showed an additional band near 460 cm^−1^, which was assigned to Cu-O vibrations. The DTD-related C-H band supported the presence of DTD in CDD NPs. The shifts in the TA bands and the Cu-O signal provided evidence for Cu–phenolic coordination.

The XRD pattern of DQ showed several sharp diffraction peaks at approximately 14.2°, 19.8°, and 21.3° (Figure 1c), which reflected its crystalline structure. These peaks were not detected in CDD NPs. The disappearance of the DQ peaks suggests that its crystallinity was substantially reduced after formulation and that DQ was present mainly in an amorphous or molecularly dispersed state. CDD NPs appeared nearly spherical in the TEM images (Figure 1d).

The surface elemental composition and Cu chemical states of CDD NPs were characterized by XPS. Signals corresponding to C, O, N, B, and Cu were observed in the survey spectrum (Figure 1e). Deconvolution of the high-resolution Cu 2p spectrum yielded components at 932.4 and 952.2 eV, which were tentatively attributed to the Cu(I) 2p_3/2_ and 2p_1/2_ states, respectively. The components at 934.4 and 954.6 eV were assigned to the corresponding Cu(II) states (Figure 1f). To further substantiate the Cu(I) assignment, complementary Cu LMM Auger analysis was performed. The spectrum exhibited a peak at 570.0 eV on the binding-energy scale (Figure S4), corresponding to a kinetic energy of 916.6 eV under Al Kα excitation. This result provides additional support for the Cu(I) assignment and when considered alongside the Cu 2p results, supports the coexistence of Cu(I) and Cu(II) in CDD NPs. Peak fitting indicated a greater relative contribution of Cu(I) in CDD NPs than in DTD-free CD NPs (Figure S5), reflecting a change in the Cu coordination and redox environment after DD NP incorporation. In conjunction with the FTIR results, this finding points to the participation of DTD in Cu coordination. The O 1s spectrum contained two components at 532.7 and 531.0 eV, assigned to O-C and O-Cu, respectively, the latter corroborating Cu-phenolic coordination (Figure 1g). Clear B 1s signals were detected in CDD NPs, whereas the corresponding signals were weak in DTD-free CD NPs (Figure S6). Because B originates exclusively from DQ, the stronger B signal indicates that DTD enhances DQ loading.

Consistent with the XPS analysis, elemental mapping showed that B, Cu, and N were broadly distributed within the CDD NP region (Figure 1h). The co-distribution of DQ-derived B and Cu supported the incorporation of DQ-containing DD NPs into the Cu@TA coordination network.

Taken together, the characterization results supported the successful construction of CDD NPs. DTD increased DQ loading through co-assembly, while its catechol groups provided coordination sites that linked DD NPs to the Cu@TA network.

### 3.2. Physiological Stability and Stimulus-Responsive Release of CDD NPs

**Figure 2:**
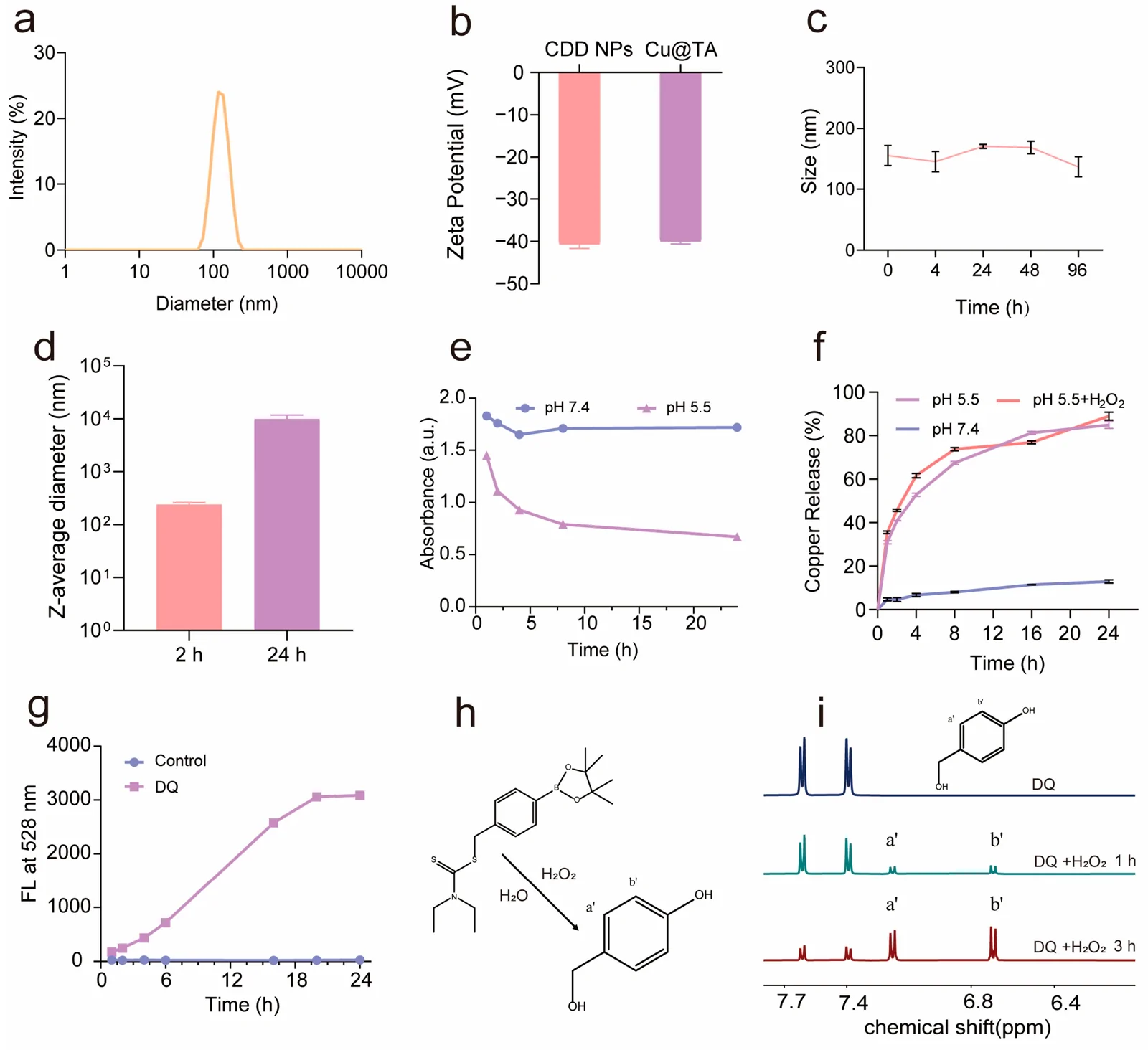
Stability and stimulus-responsive behavior of CDD NPs. (**a**) Hydrodynamic size distribution of CDD NPs; (**b**) zeta potentials of CDD NPs and Cu@TA (*n* = 3); (**c**) hydrodynamic size of CDD NPs over 96 h (*n* = 3); (**d**) Z-average diameter of CDD NPs after incubation at pH 5.5 for 2 and 24 h; (**e**) time-dependent UV–vis absorbance of CDD NPs at pH 7.4 and 5.5; (**f**) cumulative Cu release from CDD NPs at pH 7.4, pH 5.5, and pH 5.5 with H_2_O_2_; (**g**) time-dependent calcein fluorescence at 528 nm in the control and DQ groups; (**h**) schematic of H_2_O_2_-triggered DQ conversion to the aromatic hydrolysis product; (**i**) ^1^H NMR spectra of DQ before and after H_2_O_2_ treatment for 1 and 3 h.

CDD NPs exhibited a mean hydrodynamic diameter of approximately 144 nm in PBS, with a polydispersity index (PDI) of 0.193 (Figure 2a and Figure S7). In complete culture medium, the corresponding values were approximately 190 nm and 0.146, respectively (Figure S7). Their ζ-potential was −40.6 mV, close to the −39.9 mV measured for Cu@TA (Figure 2b). The large negative surface charge was consistent with electrostatic stabilization of the dispersion. Their hydrodynamic diameter showed modest variation over 96 h without a progressive increase (Figure 2c). At pH 7.4, the UV–vis absorbance also changed little during the 24 h incubation period (Figure 2e). CDD NPs therefore remained well dispersed and showed no marked spectral change under the tested conditions.

In acidic environment, protonation of the phenolic groups in TA and the catechol groups in DTD can weaken Cu–polyphenol coordination. The response observed at pH 5.5 is most relevant to CDD NP disassembly in endosomes and lysosomes after cellular uptake; the milder extracellular acidity of tumors may also facilitate this process [34,35].

At pH 5.5, the Z-average diameter increased markedly between 2 and 24 h (Figure 2d), consistent with loss of colloidal stability and aggregation. UV–vis absorbance decreased over time at pH 5.5 but changed little at pH 7.4 (Figure 2e). Photographs of the dispersions under the indicated conditions are provided in Supplementary Figure S8. Cu release remained limited at pH 7.4 and increased at pH 5.5 (Figure 2f). H_2_O_2_ accelerated the initial release at pH 5.5, although the two acidic groups reached similar cumulative release levels by 24 h. These results were consistent with acid-induced weakening of the Cu–polyphenol network.

To assess release under mildly acidic conditions relevant to the extracellular tumor microenvironment, an additional experiment was conducted at pH 6.5. Under this condition, cumulative Cu release reached approximately 57% after 30 h (Figure S9), indicating that appreciable release was not restricted to pH 5.5. The faster release under acidic conditions was consistent with weakening of Cu–polyphenol coordination and destabilization of the network. The response at pH 5.5 is particularly relevant to the endosomal and lysosomal compartments encountered after cellular uptake.

The H_2_O_2_-triggered conversion of free DQ was examined using the calcein–Cu^2+^ competition assay (Figure 2g and Figure S10). Binding of Cu^2+^ quenched the fluorescence of calcein, whereas DTC produced from DQ upon H_2_O_2_ treatment sequestered Cu^2+^ as Cu(DTC)_2_, resulting in fluorescence recovery. Calcein fluorescence increased over time in the DQ group, whereas the control remained nearly unchanged (Figure 2g). The reaction scheme depicts the formation of 4-hydroxybenzyl alcohol following H_2_O_2_ treatment and reaction with water (Figure 2h). In the aromatic region of the ^1^H NMR spectra, the original DQ signals decreased, and new signals assigned to this product appeared after 1 h and became more prominent after 3 h (Figure 2i). The fluorescence and NMR results support H_2_O_2_-triggered DQ conversion in a cell-free system, but do not directly establish intracellular Cu(DTC)_2_ formation.

### 3.3. H_2_O_2_-Dependent Oxidative Activity and GSH Depletion

**Figure 3:**
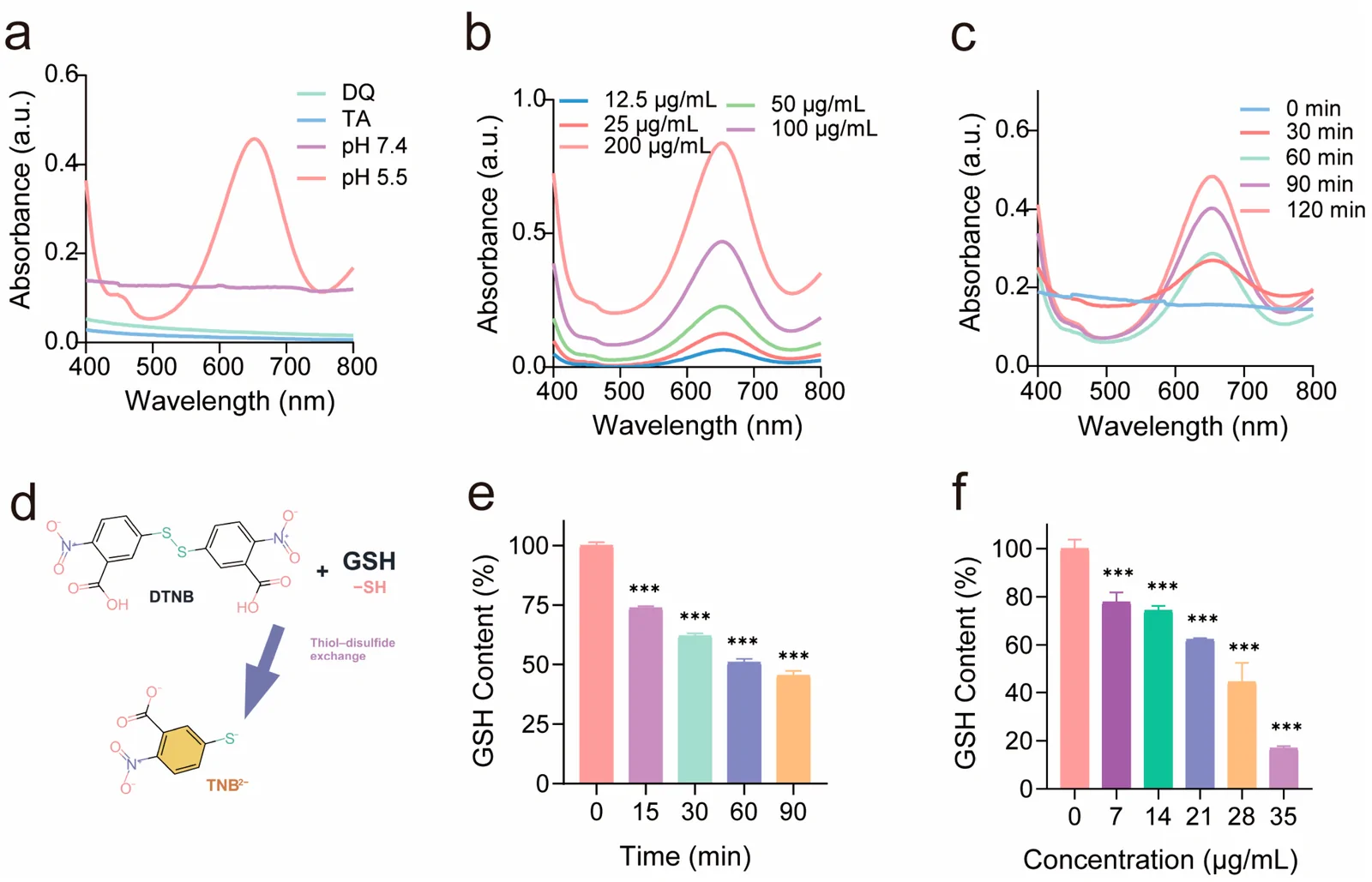
H_2_O_2_-dependent TMB oxidation and GSH depletion by CDD NPs. (**a**) UV–vis spectra of oxidized TMB in the indicated groups; (**b**,**c**) concentration and time-dependent TMB oxidation by CDD NPs; (**d**) schematic of the DTNB assay; (**e**,**f**) time- and concentration-dependent GSH depletion by CDD NPs (*n* = 3). Data are presented as mean ± SD (*n* = 3). Statistical significance was set as follows: *** *p* < 0.001, ns: not significant. *p* value was determined by one-way ANOVA test.

Copper-mediated Fenton-like chemistry is a proposed source of oxidative activity in chemodynamic therapy (CDT). TMB was used as a colorimetric indicator of this reaction because its oxidation produces blue oxTMB with a characteristic absorption peak at 652 nm [36]. In the presence of H_2_O_2_, DQ and TA alone produced little TMB oxidation. CDD NPs also showed weak activity at pH 7.4; a clear absorption peak at 652 nm appeared at pH 5.5 (Figure 3a). The peak intensity increased across the CDD NP concentration range of 12.5–200 μg/mL and continued to rise over 120 min (Figure 3b,c). The stronger response at pH 5.5 was consistent with acid-triggered Cu release and increased Fenton-like conversion of H_2_O_2_ [37,38]. Intracellular GSH can scavenge ROS, including •OH, and thereby attenuate the efficacy of CDT. In copper-based systems, Cu^+^ reacts with H_2_O_2_ through a Fenton-like reaction to generate •OH and is oxidized to Cu^2+^.

Copper redox cycling provides a possible explanation for GSH consumption. Reduction of Cu^2+^ by GSH can produce glutathione disulfide (GSSG) and Cu^+^, which may participate in further H_2_O_2_ activation. Residual GSH in reaction mixtures containing CDD NPs was measured with DTNB [39,40]. Free GSH reacts with DTNB to release yellow 5-thio-2-nitrobenzoate (TNB), detected by its characteristic absorption at 412 nm (Figure 3d) [41].

GSH declined steadily during incubation with CDD NPs, and approximately 45% remained after 90 min (Figure 3e). Increasing the CDD NP concentration produced a larger decrease; at 35 μg mL^−1^, residual GSH was about 17% (Figure 3f). Neither TA nor DQ appreciably altered the GSH level at pH 7.4 (Figure S11), suggesting weak direct reactivity of these components with GSH. The pronounced loss caused by CDD NPs was consistent with Cu-mediated GSH consumption.

### 3.4. In Vitro Anticancer Activity and Cellular Uptake

**Figure 4:**
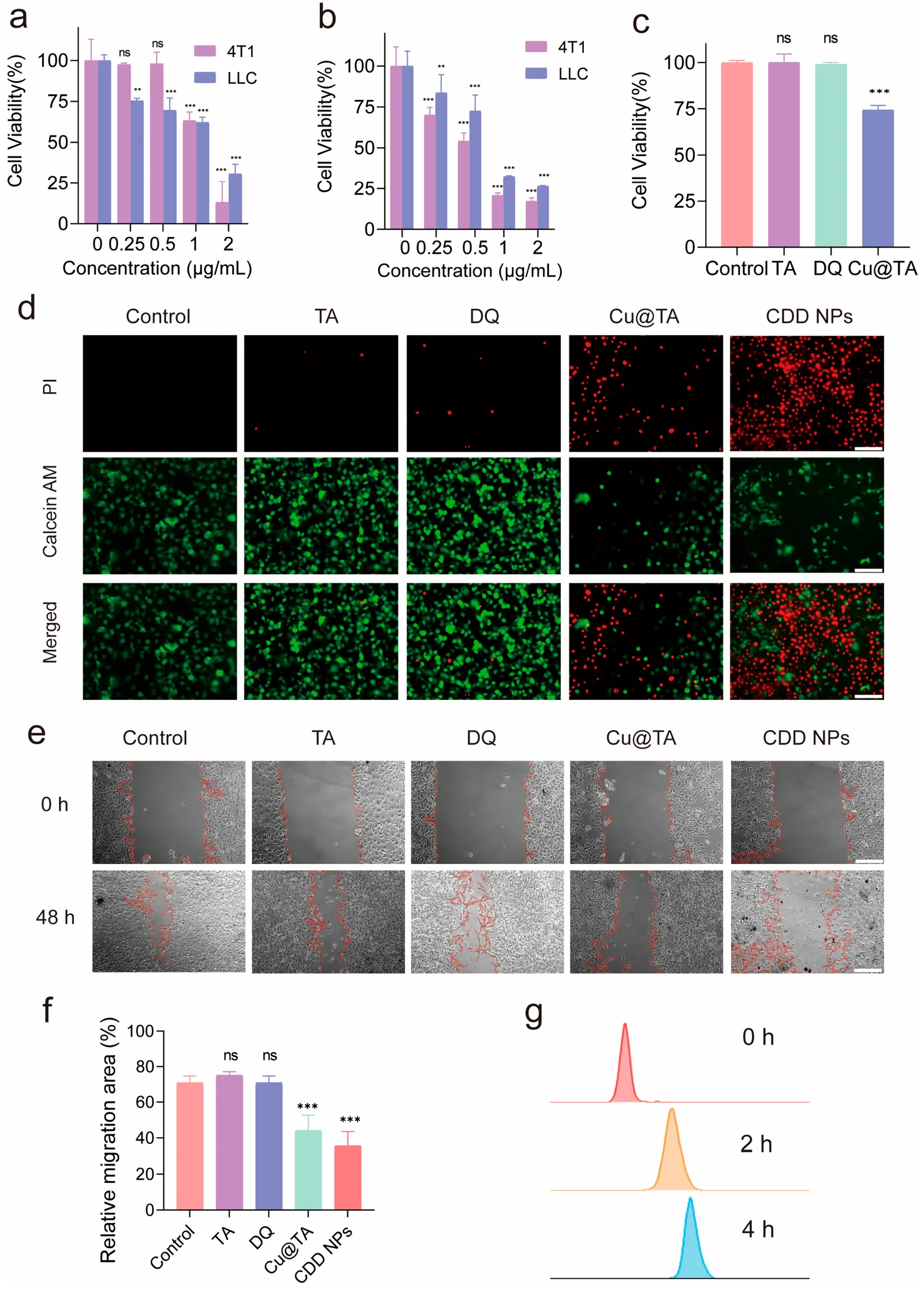
In vitro anticancer activity and cellular uptake of CDD NPs. (**a**,**b**) Viability of 4T1 and LLC cells following exposure to CDD NPs for 24 h (**a**) and 48 h (**b**) (*n* = 3); (**c**) viability of 4T1 cells in the control, TA, DQ, and Cu@TA groups (*n* = 3); (**d**) calcein-AM and PI fluorescence images of 4T1 cells in the control, TA, DQ, Cu@TA, and CDD NP groups (scale bar, 100 μm); (**e**) representative wound-healing images at 0 and 48 h (scale bar, 100 μm); (**f**) relative migration area calculated from the images in panel (*n* = 3); (**g**) flow cytometric analysis of CDD NP uptake at 0, 2, and 4 h. Data are presented as mean ± SD (*n* = 3). Statistical significance was set as follows: ** *p* < 0.01, *** *p* < 0.001, ns: not significant. *p* value was determined by one-way ANOVA test.

CDD NPs reduced the viability of both murine 4T1 cells and LLC cells in a concentration-dependent manner, as determined by the CCK-8 assay. After 24 h, approximately 13% of 4T1 cells and 31% of LLC cells remained viable at 2 μg mL^−1^ (Figure 4a). Cell viability remained low after 48 h, reaching approximately 17% and 27%, respectively, at the same concentration (Figure 4b). The effect of prolonged exposure was clearer at lower concentrations. At 0.25 μg mL^−1^, 4T1 cell viability fell from approximately 98% at 24 h to 70% at 48 h; at 1 μg mL^−1^, it fell from 63% to 21%.

TA and DQ alone had little effect on cell viability under the tested conditions; Cu@TA reduced it to approximately 74% (Figure 4c). The substantially greater reduction caused by CDD NPs suggests that Cu@TA alone did not account for the full response. In the complete construct, Cu-mediated redox chemistry may have acted alongside H_2_O_2_-responsive activation of DQ.

After 24 h of treatment, calcein-AM/PI staining showed a pattern consistent with the CCK-8 results (Figure 4d). The control and TA groups remained predominantly green, and DQ produced little additional PI signal. Without an added Cu source, formation of cytotoxic Cu(DTC)_2_ from DQ-derived DTC may have been limited. Cu@TA produced a moderate increase in red fluorescence, whereas CDD NPs caused the most pronounced shift from green to red. DTD increased DQ incorporation and linked DD NPs to Cu@TA, retaining DQ and Cu within the same formulation. H_2_O_2_-responsive DQ activation could make DTC available for copper complexation. The stronger cytotoxic response may therefore involve both prodrug activation and copper-associated oxidative stress. Because calcein-AM/PI staining cannot distinguish specific cell-death pathways, apoptosis and copper-dependent cell death were examined in subsequent experiments.

Because CDD NPs caused a pronounced loss of 4T1 cell viability, their effect on the recovery of a scratched cell monolayer was examined next. After 24 h, the scratches in the control, TA, and DQ groups had narrowed substantially; wider cell-free gaps remained after Cu@TA or CDD NP treatment (Figure S12). At 48 h, Cu@TA continued to delay wound closure, while the CDD NP group retained the widest gap (Figure 4e). Quantification showed the lowest relative migration area in the CDD NP group, followed by Cu@TA (Figure 4f). These results indicate impaired wound closure but do not establish a migration-specific inhibitory effect.

To evaluate cell association relevant to the proposed intracellular activation, the uptake of CDD NPs by 4T1 cells was examined by flow cytometry. In the flow-cytometry histograms, the fluorescence peak shifted clearly to the right after 2 h and moved further at 4 h (Figure 4g). The progressive increase in cell-associated fluorescence was consistent with time-dependent uptake, although flow cytometry alone does not distinguish internalized particles from particles bound to the cell surface. Once internalized, CDD NPs could encounter acidic endolysosomal compartments and intracellular H_2_O_2_, which could favor prodrug activation and copper release.

### 3.5. Intracellular Oxidative Stress and Apoptosis

**Figure 5:**
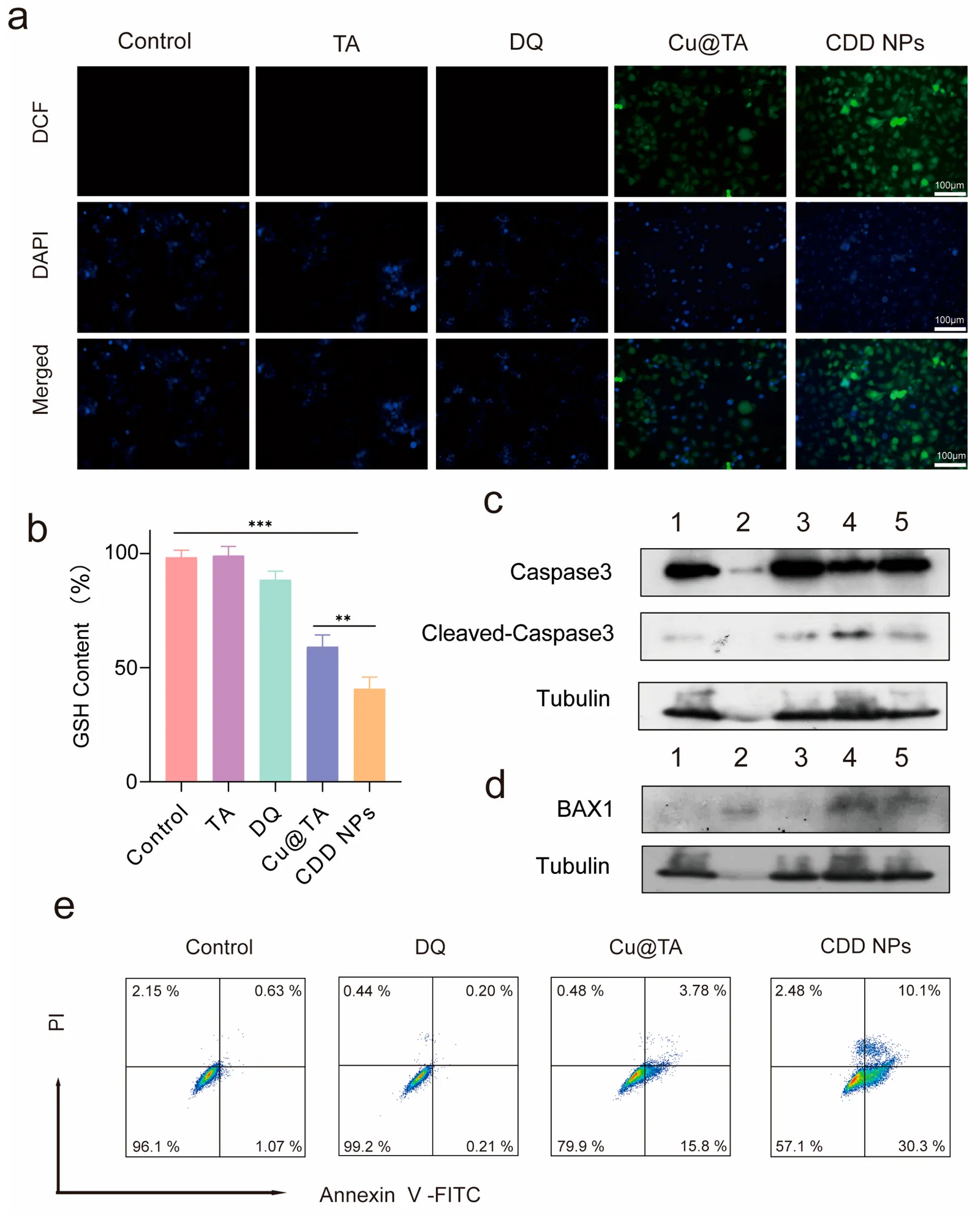
Intracellular oxidative stress and apoptosis induced by CDD NPs. (**a**) DCF fluorescence indicating intracellular ROS levels in 4T1 cells (scale bar, 100 μm); (**b**) relative intracellular GSH content (*n* = 3); (**c**,**d**) Western blot analysis of caspase-3, cleaved caspase-3, and BAX 1, with tubulin as the loading control; in (**c**,**d**), lanes 1–5 represent control, CDD NPs (20 μg mL^−1^), DQ, CDD NPs (10 μg mL^−1^), and Cu@TA, respectively. (**e**) Annexin V-FITC/PI profiles of 4T1 cells exposed to PBS, DQ, Cu@TA, or CDD NPs for 16 h. Data are presented as mean ± SD (*n* = 3). Statistical significance was set as follows: ** *p* < 0.01, *** *p* < 0.001, ns: not significant. *p* value was determined by one-way ANOVA test.

ROS accumulation in 4T1 cells was monitored with fluorescent 2′,7′-dichlorofluorescein (DCF). Oxidation of the intracellularly deacetylated probe by ROS produces green fluorescence [42]. Only weak fluorescence was detected in the control, TA, and DQ groups, whereas a clear signal was observed in cells treated with Cu@TA. CDD NP treatment produced the highest fluorescence intensity (Figure 5a and Figure S13). Together with GSH depletion, the increased fluorescence supported greater intracellular oxidative stress after CDD NP treatment. DCFH-DA is nonspecific and does not identify individual ROS.

As a major intracellular antioxidant, GSH plays an important role in maintaining cellular redox balance [43]. We therefore examined the effects of the different treatments on intracellular GSH levels [43]. TA caused no obvious change in GSH content, while DQ produced only a slight decrease. Intracellular GSH decreased to approximately 54% and 40% of the control after Cu@TA and CDD NP treatment, respectively (Figure 5b). The greater GSH depletion induced by CDD NPs may reflect copper-associated redox reactions and reactions of GSH with products of DQ activation. The lower GSH level could reduce cellular antioxidant capacity.

In addition, Annexin V-FITC and PI fluorescence signals were measured by flow cytometry to evaluate apoptosis. Viable cells comprised 96.1% and 99.2% of the populations exposed to PBS and DQ, respectively. The total Annexin V-positive fractions increased to 19.6% and 40.4% following Cu@TA and CDD NP treatment, respectively, supporting the induction of apoptosis (Figure 5e). In the CDD NP group, 10.1% of cells were Annexin V+/PI+, consistent with phosphatidylserine exposure and loss of membrane integrity. This quadrant is not specific to apoptosis and cannot by itself identify additional pathways such as cuproptosis [44].

To further investigate whether apoptosis was involved in CDD NP-induced cell death, apoptosis-related proteins were examined at the molecular level. Caspase-3 is synthesized as an inactive proenzyme. During apoptosis, proteolytic processing generates the p17 and p12 subunits, which assemble into the active enzyme that cleaves downstream cellular substrates [45]. Thus, cleaved caspase-3 is a more direct marker of caspase-3 activation than total caspase-3. Western blotting showed increased cleaved caspase-3 and the pro-apoptotic protein BAX after Cu@TA and CDD NP treatment (Figure 5c,d). Compared with the 10 μg mL^−1^ group, CDD NPs at 20 μg mL^−1^ produced weaker caspase-3 and cleaved caspase-3 signals. One possible explanation is earlier caspase-3 activation followed by degradation of the cleaved protein before sampling. A previous study reported that enzymatically active caspase-3 can undergo intracellular degradation [45]. These changes were consistent with the Annexin V-FITC/PI results and supported a contribution from caspase-3-associated apoptosis to CDD NP-induced cell death.

The increased DCF fluorescence and decreased GSH content supported oxidative stress in CDD NP-treated 4T1 cells. Annexin V/PI staining and caspase-3 cleavage supported an apoptotic contribution.

### 3.6. CDD NPs Induce Copper-Dependent Cell Death

**Figure 6:**
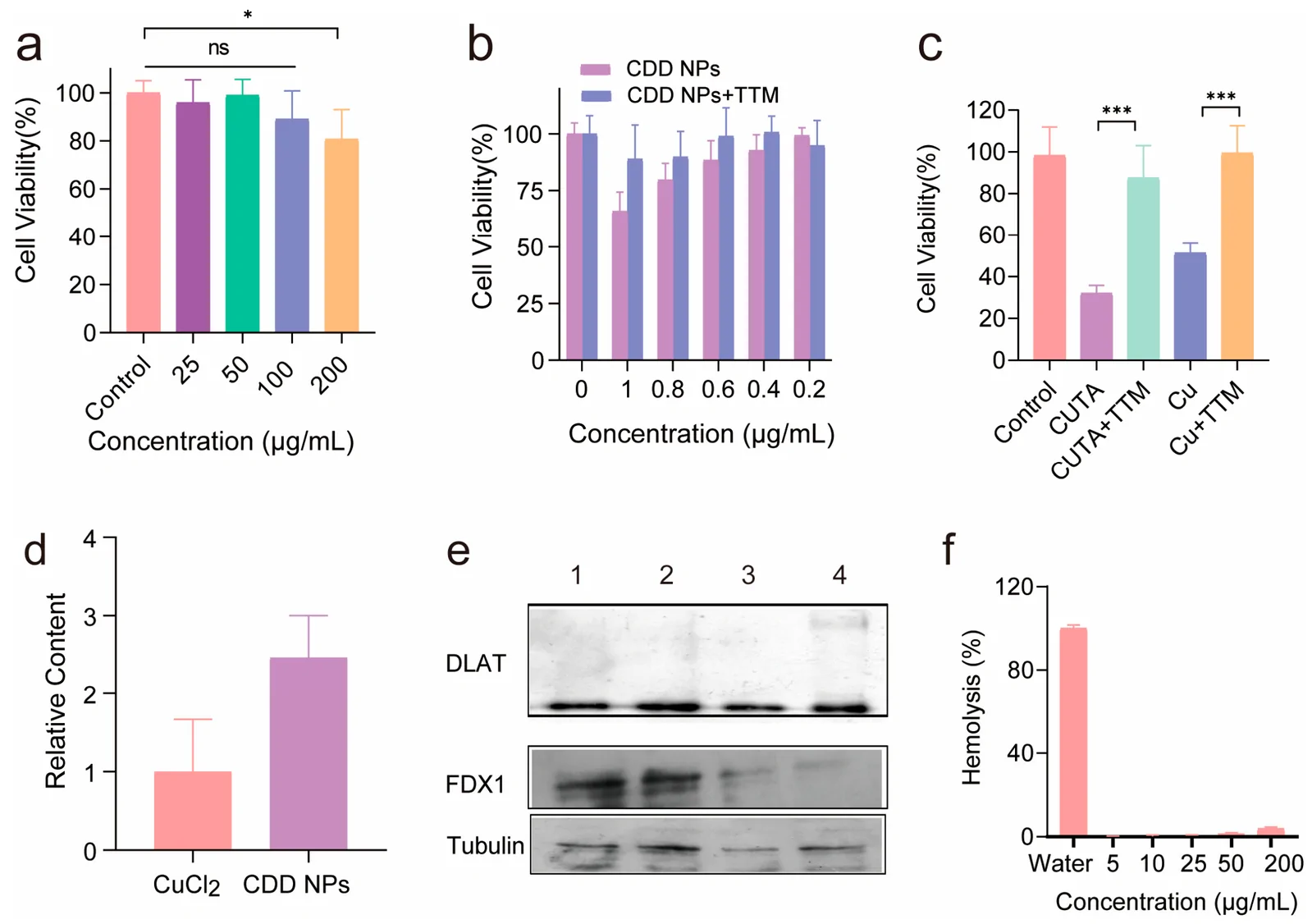
Copper accumulation and copper-dependent cell death in 4T1 cells. (**a**) Effect of TTM concentration on 4T1 cell viability (*n* = 3); (**b**) viability of CDD NP-treated cells in the presence or absence of TTM; (**c**) effects of TTM on Cu@TA and Cu-induced cytotoxicity (*n* = 3); (**d**) relative intracellular Cu content after treatment with CuCl_2_ or CDD NPs (*n* = 3); (**e**) Western blot analysis of DLAT and FDX1, with tubulin as the loading control. Lanes 1–4 represent Control, DQ, Cu@TA, and CDD NPs, respectively. (**f**) Hemolysis rates after incubation with CDD NPs at 5, 10, 25, 50, and 200 μg/mL (*n* = 3). Statistical significance was set as follows: * *p* < 0.05, *** *p* < 0.001, ns: not significant. *p* value was determined by one-way ANOVA test.

To determine whether copper contributed to CDD NP-induced cell death, intracellular Cu was measured by ICP. CDD NP-treated cells accumulated approximately 2.5 times as much Cu as CuCl_2_-treated cells (Figure 6d). During cuproptosis, excess Cu interacts directly with lipoylated proteins of the tricarboxylic acid cycle. The resulting proteotoxic stress involves aggregation of these proteins and a reduction in iron–sulfur cluster proteins [1,38]. The elevated copper content therefore provided a basis for further examining cuproptosis in CDD NP-treated cells.

To determine whether copper contributed to CDD NP-induced cell death, rescue experiments were performed using the copper chelator TTM. TTM caused little loss of cell viability at low-to-moderate concentrations, and 20 μM was selected for subsequent experiments (Figure 6a). TTM increased the viability of CDD NP-treated cells, showing that copper contributed to their cytotoxicity (Figure 6b). A similar rescue was observed for Cu@TA and free Cu: cell viability increased from approximately 32% to 88% in the Cu@TA group and from 51% to nearly 100% in the Cu group after TTM treatment (Figure 6c). These controls linked the rescue effect of TTM to copper chelation, consistent with previous reports that TTM prevents copper-induced cell death [1]. However, TTM failed to rescue cells at higher CDD NP concentrations (Figure S14). This may result from the limited capacity of a fixed TTM concentration and increased formation of cytotoxic Cu(DTC)_2_ following DQ activation, which may not be fully reversed by TTM. Additional rescue experiments were then performed using ferrostatin-1 (Fer-1) and the pan-caspase inhibitor Z-VAD-FMK to assess the contributions of other cell-death pathways. Fer-1 did not improve cell viability following CDD NP treatment (Figure S15a), whereas Z-VAD-FMK partially restored viability (Figure S15b), supporting a contribution from caspase-dependent apoptosis. The lack of Fer-1 rescue suggests that ferroptosis was not a major contributor under the tested conditions.

The TTM rescue and increased intracellular copper prompted examination of FDX1 and DLAT by Western blotting. FDX1 controls mitochondrial protein lipoylation and plays an essential role in cuproptosis. Excess copper binds lipoylated DLAT and promotes its oligomerization, leading to proteotoxic stress [46,47]. CDD NP treatment reduced FDX1 expression and increased oligomerized DLAT, while total DLAT remained largely unchanged (Figure 6e). The combined evidence from copper accumulation, TTM rescue, and protein changes supported copper-dependent cell death and suggested a possible contribution of cuproptosis.

To assess the preliminary in vitro safety and selectivity of CDD NPs, their cytotoxicity toward nonmalignant NIH 3T3 fibroblasts was compared with that toward 4T1 tumor cells. At 2 μg/mL, NIH 3T3 cell viability remained approximately 83%, markedly higher than that of 4T1 cells under identical conditions (Figure S16). Hemocompatibility was further evaluated using a red blood cell hemolysis assay. The hemolysis rates were 0.18%, 0.27%, 0.50%, 1.37%, and 3.88% at CDD NP concentrations of 5, 10, 25, 50, and 200 μg/mL, respectively (Figure 6f). Hemolysis remained below 2% at concentrations up to 50 μg/mL and below 5% throughout the tested concentration range. These findings support the preliminary biocompatibility of CDD NPs under the tested in vitro conditions.

## 4. Discussion

In summary, we developed a copper-containing, ROS-responsive nanoprodrug, termed CDD NPs, by coating DTD/DQ co-assembled DD NPs with a Cu@TA coordination network. DTD promoted the incorporation of hydrophobic DQ into DD NPs, while its catechol groups contributed to the formation of the Cu@TA network. Stimulus-responsive prodrug assemblies have previously been used for controlled release [25], and metal–polyphenol networks have served as pH-responsive coatings [28]. In CDD NPs, DTD links these two processes through its amphiphilic structure and copper-binding catechol groups. The evidence supports DTD as a coordination-active component at the interface between the DQ-containing core and the Cu@TA network. Its contribution therefore concerns formulation architecture as well as loading.

The key advantage of CDD NPs is the integration of prodrug co-assembly and copper coordination through DTD. DTD promotes DQ incorporation while connecting the co-assembled core to the Cu@TA network, with DQ loading approximately 1.75-fold that of the DTD-free control. CDD NPs contained 6.70 ± 0.17 wt% DQ and 26.71 ± 0.17 wt% copper. Their hydrodynamic diameter was approximately 144 nm in PBS, within the range reported for Cu(I) nanoparticles (86 nm) [48], ECPCP (109.4 nm) [37], and PEG-DOX@Cu (160 nm) [49]. Compared with DSF/Cu-based formulations, CDD NPs also exhibited a smaller reported hydrodynamic diameter than CLDCu (186.9 nm) [50] and DSF-CuS@TA (208.7 nm) [51]. PEG-DOX@Cu contained 18.6 wt% [49] doxorubicin and 4.2 wt% copper, whereas CLDCu contained 2.97 wt% copper [50]. The copper mass fraction of CDD NPs was therefore approximately 6.4-fold that of PEG-DOX@Cu and 9.0-fold that of CLDCu, providing greater copper-carrying capacity per unit formulation mass. Collectively, these comparisons highlight the ability of the DTD-mediated architecture to integrate prodrug incorporation and a substantial copper payload within a relatively compact nanostructure. Different payloads and characterization conditions nevertheless limit direct comparisons of drug-loading performance and therapeutic efficacy.

CDD NPs combine acid-sensitive copper release with H_2_O_2_-responsive prodrug activation. Without added H_2_O_2_, cumulative copper release reached approximately 84% at pH 5.5 within 24 h, compared with approximately 13% at pH 7.4. This represents an approximately 6.5-fold difference in cumulative release between acidic and neutral conditions. For comparison, PEG-DOX@Cu released approximately 83% of its copper at pH 5.0 after 52 h [49], whereas TCCHA released 67.5% under the same pH after 48 h [52]. CDD NPs thus achieved a comparable or higher cumulative release at a shorter reported endpoint and under less acidic conditions. Porous hollow copper iron oxide nanoparticles released 21.27% of their copper at pH 5.5 within 24 h [53], below the fraction observed for CDD NPs.

The cellular findings suggest that copper-dependent toxicity accompanies oxidative stress and apoptotic signaling. Partial TTM rescue supports copper involvement, while increased oligomerized DLAT is consistent with the protein aggregation associated with cuproptosis [1]. Nevertheless, these observations do not establish cuproptosis as the dominant death pathway. The absence of TTM rescue at higher CDD NP concentrations may reflect insufficient chelation or additional effects following prodrug activation; these possibilities remain unresolved. Combining genetic perturbations targeting FDX1 or protein lipoylation with pathway-selective rescue experiments would clarify the relative contributions of cuproptosis and apoptosis.

The main value of this design lies in integrating the co-assembly component into the copper coordination network, thereby retaining DQ and its copper reaction partner within the same nanoparticle. This architecture provides an experimental basis for further investigation of integrated prodrug–coordination nanomedicines.

A central limitation of this study is that all biological evaluations were performed in vitro, and the mechanistic analysis focused primarily on 4T1 cells. The present findings therefore do not establish in vivo antitumor therapeutic efficacy, preferential tumor accumulation, or systemic safety. Although the NIH 3T3 cytotoxicity and hemolysis assays provide preliminary information on in vitro biocompatibility, they cannot predict tolerability in animals. Future studies using appropriate animal tumor models are needed to assess tumor accumulation, therapeutic efficacy, biodistribution, systemic copper-related toxicity, and the longer-term degradation and clearance of the metal–polyphenol components. These evaluations will be necessary to determine whether the observed cellular effects translate into therapeutic benefit with acceptable safety in vivo.

## Data Availability

The data and materials generated and analyzed during this paper can be obtained from the corresponding authors upon reasonable request.

## Supplementary Materials

The following supporting information can be downloaded at: https://www.mdpi.com/article/10.3390/nano16201301/s1, Figure S1: ^1^H NMR data of DQ in CDCl_3_; Figure S2: ^13^C NMR spectrum for DQ recorded in CDCl_3_; Figure S3: ^1^H NMR spectra of TK and DTD in DMSO-d_6_; Figure S4: Cu LMM Auger spectrum of CDD NPs; Figure S5: High-resolution Cu 2p XPS spectrum of CD NPs; Figure S6: High-resolution B 1s XPS spectra of (a) CDD NPs and (b) CD NPs; Figure S7: Hydrodynamic diameter and polydispersity index (PDI) of CDD NPs in complete culture medium and PBS; Figure S8: Representative photographs of CDD NP dispersions after incubation at pH 7.4, pH 5.5, and pH 5.5 (from left to right) in the presence of H_2_O_2_; Figure S9: Cumulative Cu release from CDD NPs at pH 6.5; Figure S10: ESI-MS spectrum of the reaction mixture following the DQ competition assay; Figure S11: GSH depletion by TA and DQ; Figure S12: Wound-healing images at 24 h; Figure S13: Quantitative analysis of ROS production in different treatment groups; Figure S14: Viability of cells treated with CDD NPs (blue) with or without (purple) TTM; Figure S15: Viability of cells treated with CDD NPs with or without Fer-1 (a) and Z-VAD (b); Figure S16: The cell viability of NIH 3T3 and 4T1 cells after being treated with CDD NPs. Table S1. DQ loading content and copper content of CDD NPs. Data are presented as mean ± SD (*n* = 3).
