# Supplementary material for: Dual-Responsive Nanoparticles for Prodrug Activation and Copper-Dependent Cell Death
Source: Nanomaterials (Basel). 2026 Oct 14;16(20):1301. doi: 10.3390/nano16201301 (PMC13708531; doi:10.3390/nano16201301)
Supplement: Supplementary file 1 [file nanomaterials-16-01301-s001.zip › nanomaterials-4588580-supplementary.pdf]

Supplementary Materials

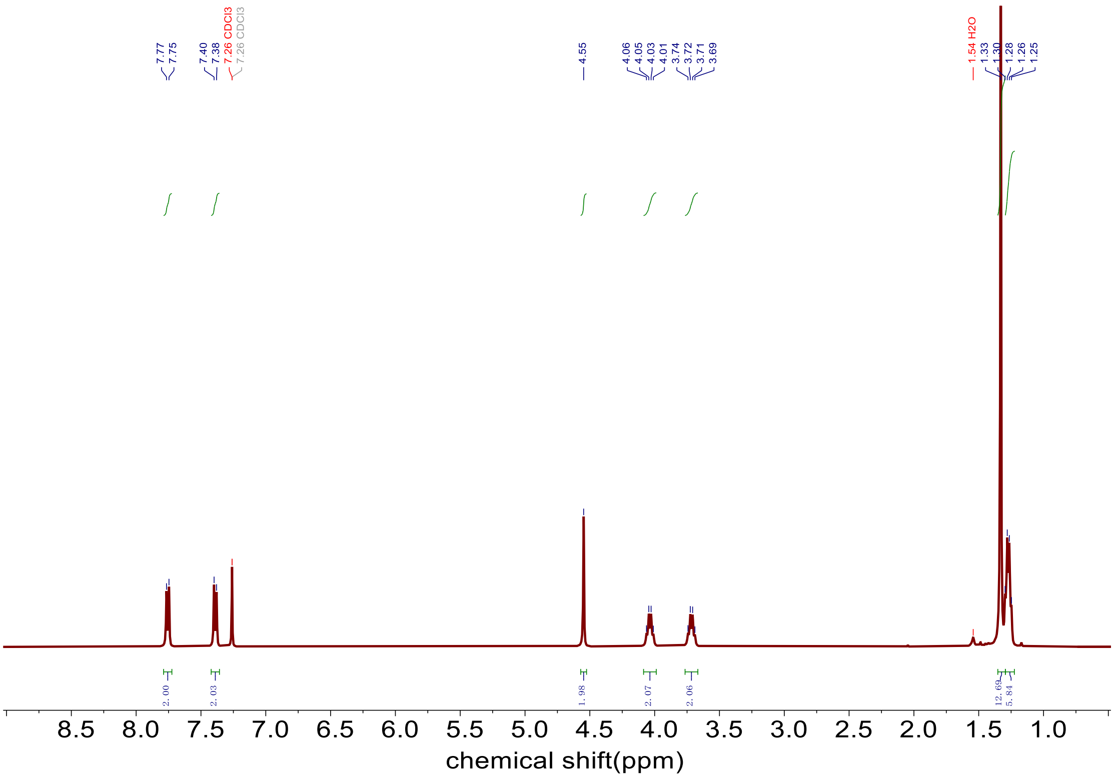

Figure S1. ^{1}H NMR spectrum of DQ in $CDCl_3$.

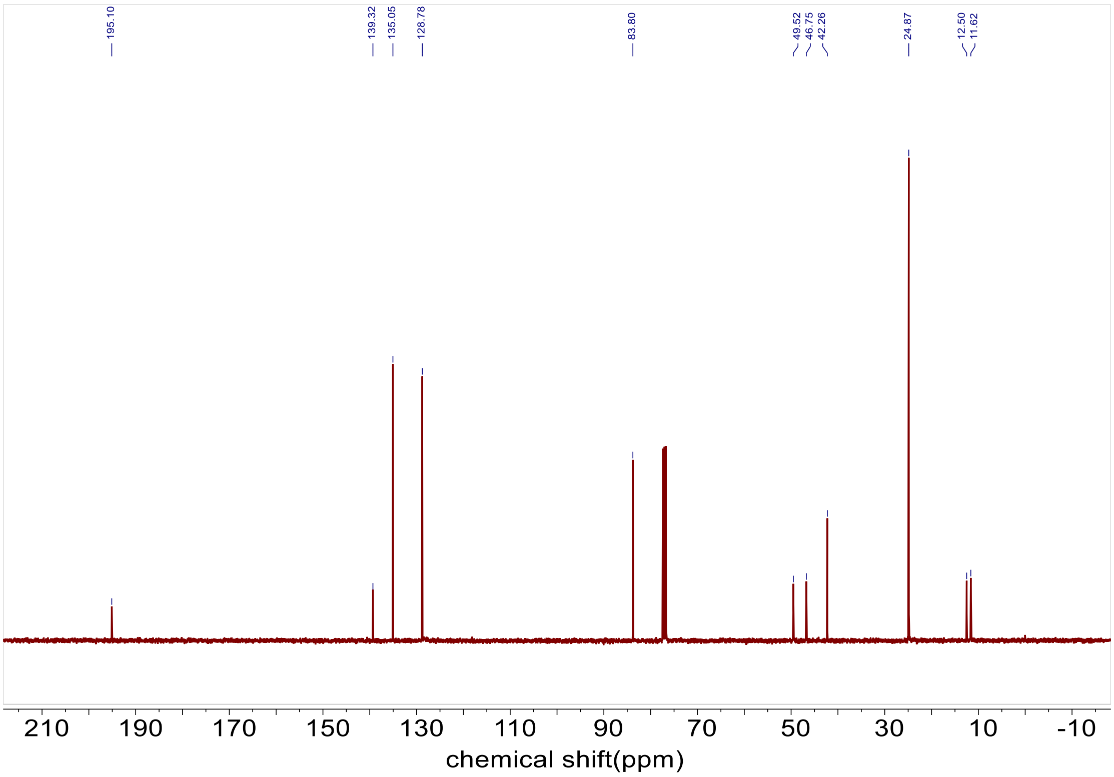

Figure S2. ^{13}C NMR spectrum of DQ in $CDCl_3$.

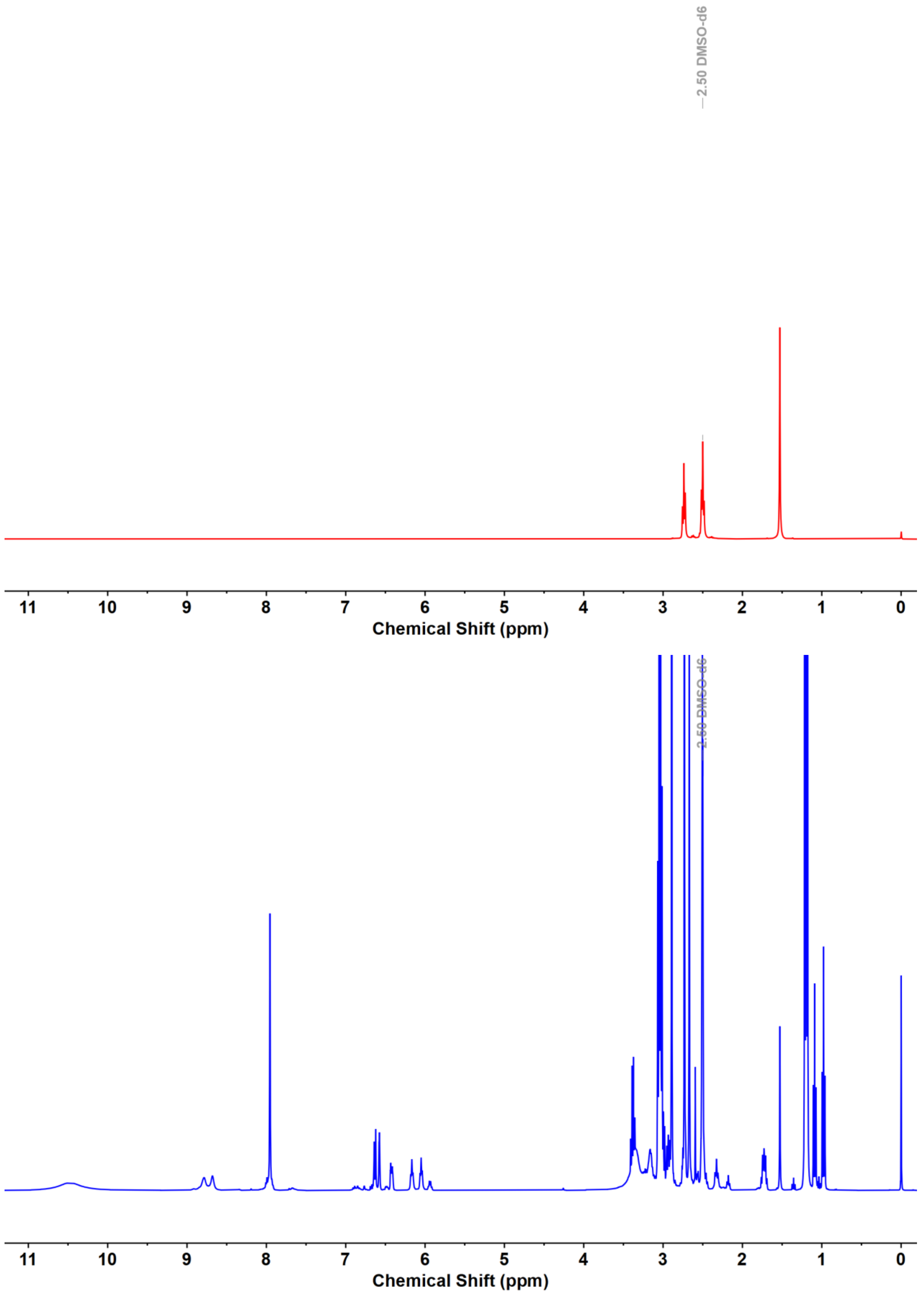

Figure S3. ^{1}H NMR spectra (Dimethyl sulfoxide-d6 as solvent) of TK and DTD.

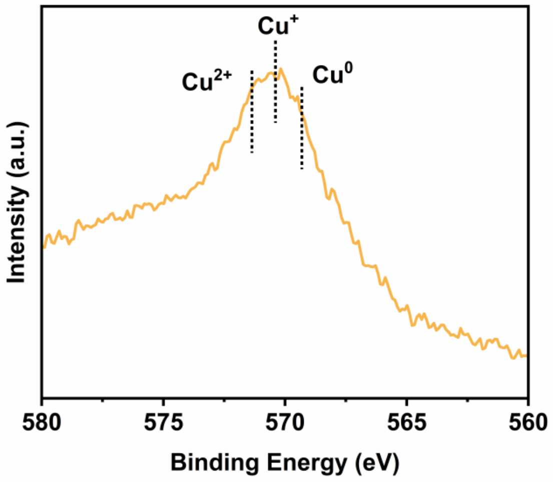

Figure S4. Cu LMM Auger spectrum of CDD NPs.

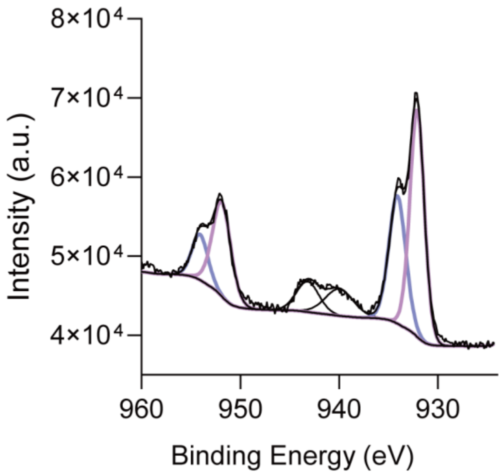

Figure S5. High-resolution Cu 2p XPS spectrum of CD NPs.

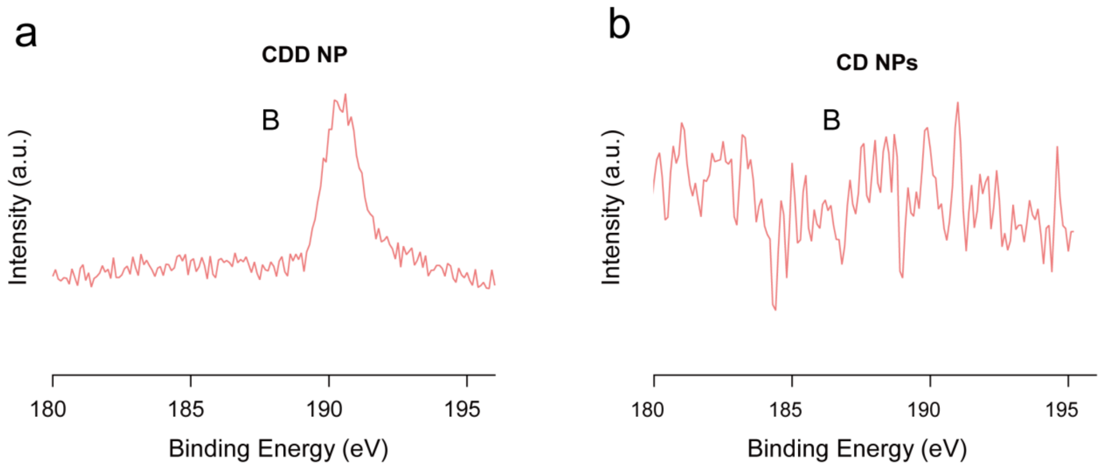

Figure S6. High-resolution B 1s XPS spectra of (a) CDD NPs and (b) CD NPs.

Sample	DQ loading content (wt%)	Copper content (wt%)
CDD NPs	6.70 ± 0.17	26.71 ± 0.17

Table S1. DQ loading content and copper content of CDD NPs. Data are presented as mean ± SD (n = 3).

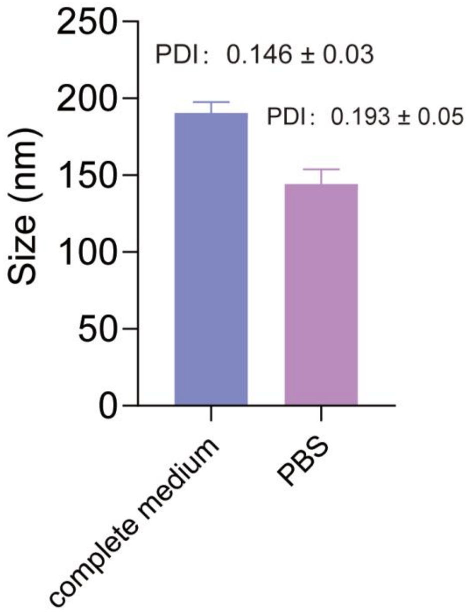

Figure S7. Hydrodynamic diameter and polydispersity index (PDI) of CDD NPs in complete culture medium and PBS. Data are presented as mean ± SD (n = 3).

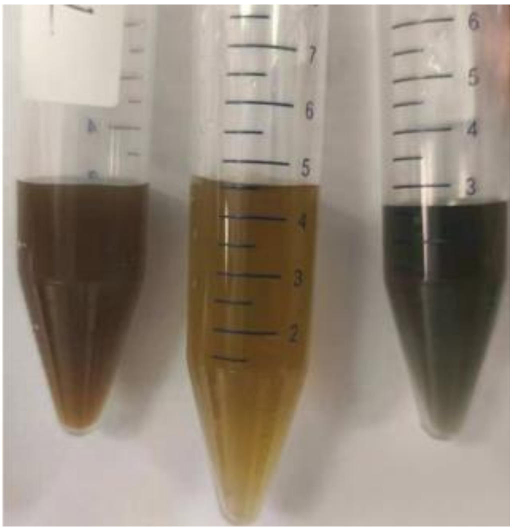

Figure S8. Representative photographs of CDD NP dispersions after incubation at pH 7.4, pH 5.5, and pH 5.5 in the presence of H_2O_2 (from left to right).

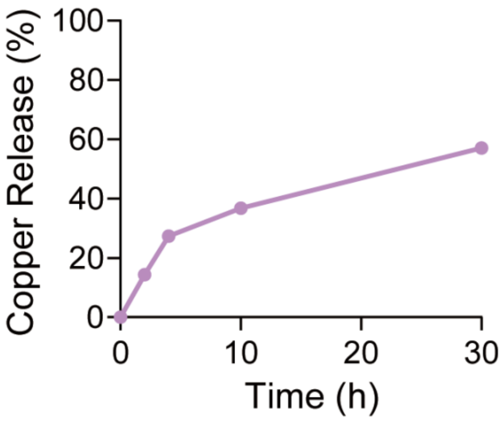

Figure S9. Cumulative Cu release from CDD NPs at pH 6.5.

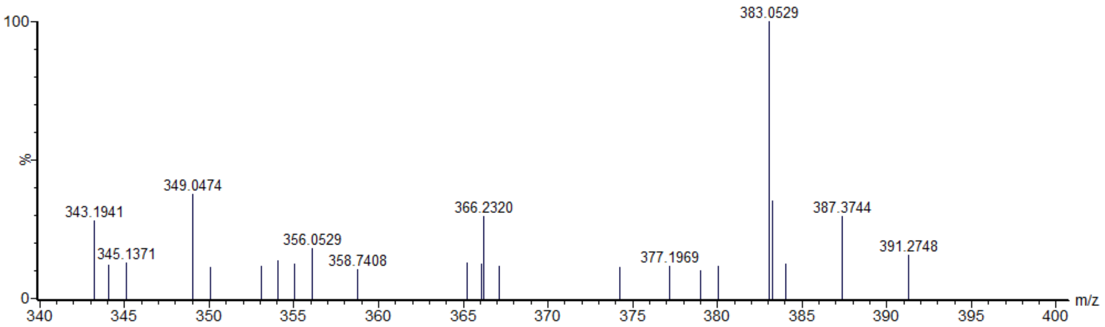

Figure S10. ESI-MS spectrum of the reaction mixture following the DQ competition assay.

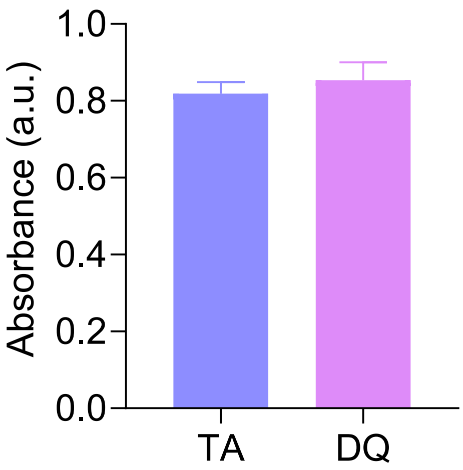

Figure S11. GSH depletion by TA and DQ.

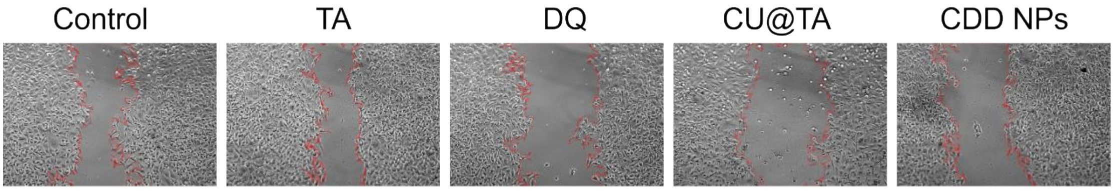

Figure S12. Wound-healing images at 24 h.

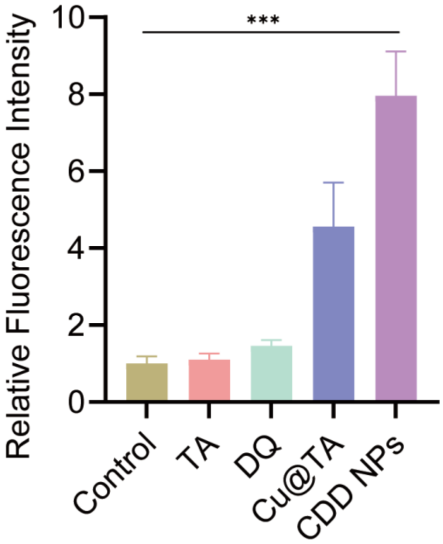

Figure S13. Quantitative analysis of ROS production in different treatment groups. Data are presented as mean ± SD (n = 3). Statistical significance was set as follows: $***p < 0.001$, ns: not significant. P value was determined by one-way ANOVA test.

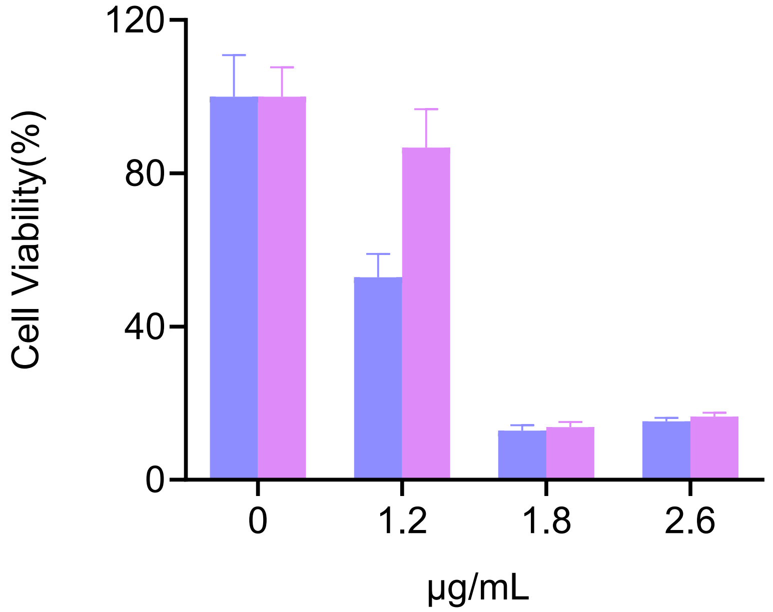

Figure S14. Viability of cells treated with CDD NPs (blue) with or without (purple) TTM.

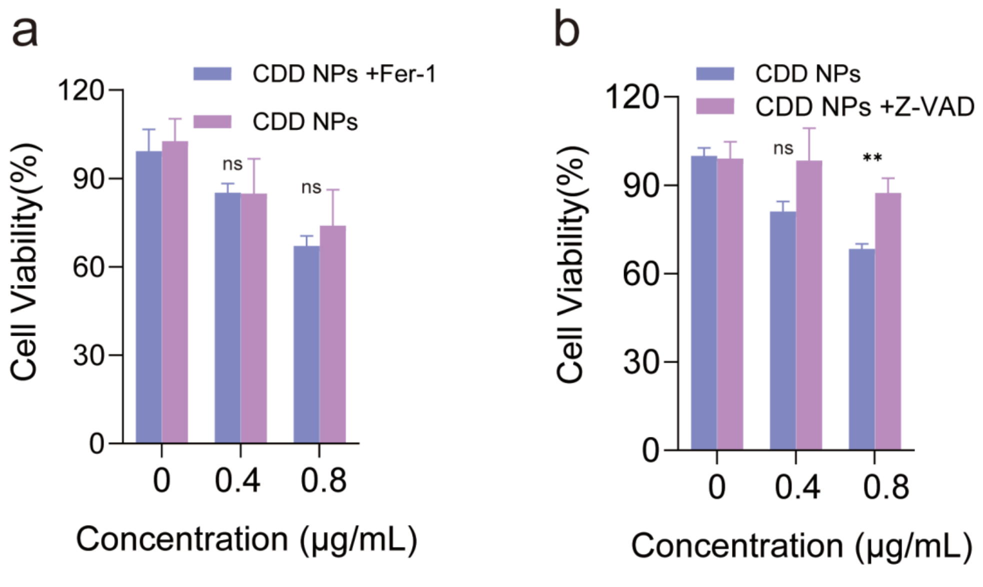

Figure S15. Viability of cells treated with CDD NPs with or without Fer-1 (a) and Z-VAD (b).

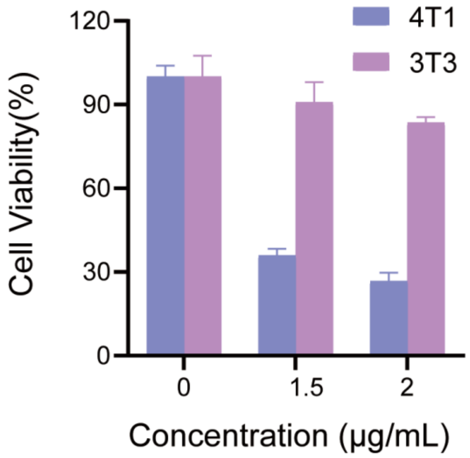

Figure S16. The cell viability of NIH 3T3 and 4T1 cells after being treated with CDD NPs.
